# Search and Coherence-Building in Intuition and Insight Problem Solving

**DOI:** 10.3389/fpsyg.2017.00827

**Published:** 2017-05-29

**Authors:** Michael Öllinger, Albrecht von Müller

**Affiliations:** ^1^Parmenides Center for the Study of ThinkingPullach, Germany; ^2^Psychological Department, Ludwig-Maximilians-Universität MünchenMunich, Germany; ^3^Philosophical Department, Ludwig-Maximilians-Universität MünchenMunich, Germany

**Keywords:** insight, intuition, binding, coherence, stage models

## Abstract

Coherence-building is a key concept for a better understanding of the underlying mechanisms of intuition and insight problem solving. There are several accounts that address certain aspects of coherence-building. However, there is still no proper framework defining the general principles of coherence-building. We propose a four-stage model of coherence-building. The first stage starts with spreading activation restricted by constraints. This dynamic is a well-defined rule based process. The second stage is characterized by detecting a coherent state. We adopted a fluency account assuming that the ease of information processing indicates the realization of a coherent state. The third stage is designated to evaluate the result of the coherence-building process and assess whether the given problem is solved or not. If the coherent state does not fit the requirements of the task, the process re-enters at stage 1. These three stages characterize intuition. For insight problem solving a fourth stage is necessary, which restructures the given representation after repeated failure, so that a new search space results. The new search space enables new coherent states. We provide a review of the most important findings, outline our model, present a large number of examples, deduce potential new paradigms and measures that might help to decipher the underlying cognitive processes.

## Introduction

During 1916 Max Wertheimer, the famous Gestaltist, and Einstein had several discussions. Wertheimer was keen to understand Einstein’s outstanding thinking. He realized that Einstein was already puzzled by apparent unanswerable questions at a very early stage, such as: “What would happen if one rode on a ray of light, or what would happen if one ran fast enough? Would the light stop to move?” Einstein felt an incoherence between the novel experimental findings at this time and the given theoretical assumptions. However, he was not able to put the single pieces together and arrange them in a new coherent picture. It was unclear how such a new picture should look like. According to Wertheimer, Einstein experienced the intuition that the common presuppositions in physics might be wrong. By that time, Einstein had the ingenious insight that the measurement of time is dependent on the applied frame of reference. By using this insight, he relaxed the existing dogmas, and eventually the single pieces became part of a coherent picture.

Wertheimer questioned that Einstein attained his great insight by the concatenation of logical operations. “Einstein did not put ready-made axioms, or mathematical formulas together.” (p. 183). He emphasized that Einstein’s progress was characterized by structural changes which were driven by overcoming the traditional understanding of physical events, time and simultaneity. Wertheimer remarked that Einstein’s thinking was often far ahead of the available mathematical apparatus.

Einstein himself reported that his thinking was not bound to words. He used mostly pictures and imagination, as his early thought experiments (Gedankenexperiment, see above) demonstrated. “I very rarely think in words at all. A thought comes, and I may try to express it in words *afterward*” (Wertheimer, 1959).

Einstein’s thinking showed how literally a new and coherent picture leads to the solution of a difficult problem.

Currently, coherence-building plays an important role within cognitive psychology. Coherence is the key concept in a great number of studies on intuition (e.g., Dorfman et al., 1996; Shirley and Langan-Fox, 1996; Bolte et al., 2003; Bolte and Goschke, 2008; Volz et al., 2008; Dehaene, 2009; Topolinski and Strack, 2009b; Zander et al., 2016) and in a few studies on insight problem solving (Metcalfe and Wiebe, 1987; Bowden and Beeman, 1998; Kounios et al., 2006).

*Intuition* can be understood as a widely unconscious process, which provides a hunch for a judgment, which is often accompanied by an affective state or gut feeling (Gigerenzer and Todd, 2001a; Kruglanski and Gigerenzer, 2011). A standard task, which demonstrates the dynamic of intuitive judgments, is the word-triads task. Mednick and Mednick (1967) introduced this task. The original task requires finding a fourth word which builds meaningful compounds with three given words (e.g., SALT, DEEP, FOAM could be associated with the word SEA resulting in three meaningful compounds such as SEA SALT, etc.). In a modified version (Bolte et al., 2003; Topolinski and Strack, 2009c) participants were asked to make quick judgments on whether a given triad was coherent or incoherent without searching for associates. Note that incoherent trials had no obvious associate (e.g., DREAM, BALL, and BOOK).

*Insight problem solving* requires participants to find the solution to a given problem. E.g., the solution of the above presented coherent triads or more difficult problems such as puzzles (Sternberg and Davidson, 1995; Jung-Beeman et al., 2004; Bowden et al., 2005; Öllinger and Knoblich, 2009; Öllinger et al., 2014). Insight problems are often characterized by the fact that they are resistant to standard solution approaches. They often require restructuring the given problem or goal representation (Ohlsson, 1984a,b, 1990, 2011; Fleck and Weisberg, 2013). Insight problem-solving goes usually beyond the information which is actually given (c.f. Bowers et al., 1990, p. 74).

Although intuition and insight are often treated as different research domains, they obviously share certain features (see below). There are only a few studies addressing both and aiming at an integrated framework (Bowers et al., 1990, 1995; Kihlstrom, 1998; Topolinski and Reber, 2010; Zander et al., 2016). In this vein, we attempt to provide an integrated view which merges both domains by rule-based coherence-building processes.

Bowers et al. (1990) seminal work on “Intuition in the context of discovery” coherence was supposed to be the key process underlying intuition and insight. Coherence results from a widely unconscious and guided search process, which converges in an integrated representation of the given information, which surpasses the threshold to consciousness.

In greater detail, the *guiding stage* is driven by spreading activation within mnemonic networks (Collins and Loftus, 1975). Those activation patterns build up to an implicit and unconscious “perception of coherence” (Bowers et al., 1990, p. 74). This tacit perception of coherence guides the thought toward a more “explicit perception in question.” It is important to note that Bowers et al. (1990) did not assume that such an implicit coherent representation is equal to the later consciously experienced coherence, but provides a fragmentary representation which could be enriched gradually by accumulating information.

Eventually, the *integrative stage* provides the result of a completed accumulation process. The activation within the network becomes so strong that it crosses the threshold to consciousness. At this stage coherence is, recognized as a hunch, which needs to be validated by an analytic validation process.

Although an exact definition of coherence was not provided, Bowers and colleagues’ experimental design elucidates its alleged characteristics, e.g., in experiment 3a Bowers and colleagues asked participants to find an unknown solution word while a list of up to 15 clue words was presented subsequently. Each clue word was associated with the unknown solution word. An example of the accumulated clues task is for instance: (1) “Times”, (2) “Inch”, (3) “Deal”, (4) “Peg”, (5) “Head”, (6) “Foot”, (7) “Dance”, (8) “Table”, (9) “Person”, (10) “Town”, (11) “Math”, (12) “Four”, (13) “Block”, (14) “Table”, (15) “Box”. The target word is “Square.”

**FIGURE 1:**
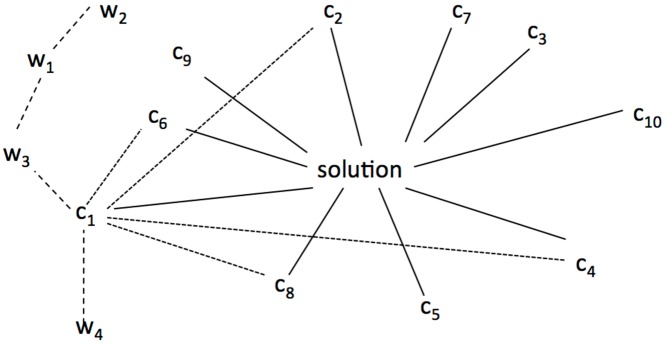
**Spreading activation and coherence.** C_1_ … C_n_ indicate clue words that were associated with the solution word. W_1_…W_n_ illustrate activated nodes which are not associated with the solution. Solid lines show associations with the solution word. Doted lines exemplarily show associations between C_1_ and other clues. Dashed lines show spreading activation between words. The length of the lines indicates the strength of the association.

One result was that participants needed up to 10 clue words to find the solution. **Figure 1** illustrates the idea of associations and spreading activation. Each clue word is associated with the unknown target word (solution).

## Limitations of Bowers Stage Model

Given the importance Bowers and colleagues’ approach, we want to draw attention to a few concerns that we have with the current model.

First, the idea of a guided accumulation process is striking, but seems underspecified and unclear. Spreading activation elicits literally unspecific neighboring nodes in the network. That means the more clues are provided, the more activity should confuse the search process. The question is what guides the process? Pure associations would not be able to guide the process, since too many unspecific associations are activated by, e.g., 10 very different clue words. That is, the potential search space would explode.

We propose that the given information activates concepts from long-term memory. Spreading activation provides a bulk of information which either belongs to the solution of the problem or not. We assume that finding a coherent representation requires constraining the search space. In the easiest case this could be attained by identifying overlapping features or meanings as in the word clue example above.

We conclude that for those problems it is necessary to have a concerted interplay between spreading activation and constraining (Ohlsson, 1990; Thagard and Verbeurgt, 1998; Thagard, 2002) the activation landscape in a goal-directed manner. More difficult problem representations require constraining the search space by prior knowledge, hypotheses or chunking of information which structures and guides the process of coherence building (implications see below).

Our second concern refers to the transition between unconscious and conscious stage is somewhat unclear. We adopt a fluency account (Topolinski and Strack, 2009a,c) which relies on the ease of the processing of the given information. We assume that a constrained activation leads to a balance state (Heider, 1946), which could easily be processed, and results in the realization of a coherent state.

**FIGURE 2:**
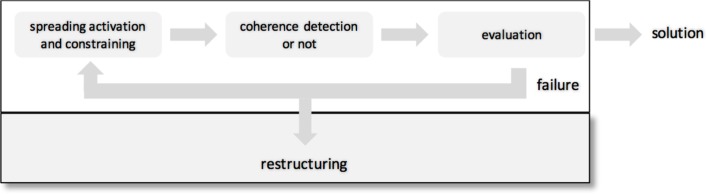
**A four-stage model of coherence-building.** The search starts in a restricted search space (white rectangle at the top). After spreading activation and constraining coherence can be detected or not. A subsequent evaluation stage validates and assesses the result of the coherence detection process. Either a solution is found or a failure occurs. After a failure the search process re-starts at stage 1 or the search space is restructured resulting in a larger search space (gray rectangle + white rectangle).

Third, the result of the integration process is a hunch or intuition which had to be validated and checked (Wallas, 1926). We propose a separate process for that and a re-entry loop, if the result is unsatisfactory or erroneous (**Figure 2**). Importantly, after repeated failure it might be necessary to restructure the search space to find a coherent state in an even larger search space. The new search space allows to integrate new information.

We hypothesize that this four-stage model allows to describe coherence-building. We further suggest that at each stage implicit and explicit processes are involved, however, the ratio between them varies to a great extent across stages. Therefore, different measures are necessary to pinpoint the underlying cognitive processes at the different stages.

In the following section, we will elaborate on the four stages by collecting evidence from different fields for each stage.

## Stage 1: Spreading Activation and Constraining

As **Figure 1** illustrates a spreading activation account is not sufficient to explain the emergence of coherence. Pure spreading activation would result in an unsynchronized activation of unrelated information which distorts the coherence building process (see *W*_ns_ in **Figure 1**).

We assume that each word activates associations (via spreading activations in the semantic network). The given clues are constraining (shaping) the search space. They are strengthening particular features of the activated concepts, and inhibiting others, at the same time. The interplay between the features of the clues, which also could be interrelated, constrains the search space until the solution word is isolated. Coherence is attained by finding the intersection of all the associations of the clue words. For the clue experiment that means that the more clues are provided, the narrower becomes the search space until the target word is isolated. The more overlapping associations the clues have, the more likely is the detection of a coherent state. For the word triads task this would explain why “coherent triads” are processed faster than “incoherent triads.” Incoherent triads share less association which constrain the search space, whereas coherent triads do.

Our argumentation is closely related to the work of Holyoak and Thagard (1995), Thagard and Verbeurgt (1998) and Thagard (2002). They provided a rule based definition of coherence. Coherence follows a constraint satisfaction process. Constraint satisfaction is an idea which was successfully applied in connectionistic models, for example to model ambiguous figure perception (McClelland et al., 1986; McClelland and Rumelhart, 1989). An illustrative example is for example a model of the Necker cube (Necker, 1832), where the nodes of one cube representation exited themselves in parallel. That leads to a stable and coherent state. The exited nodes concurrently inhibit the nodes of the alternative cube representation.

Thagard and Verbeurgt (1998, see p. 2–3 for the detailed list) stated seven computational principles that define coherence:

(1) Elements are representations (e.g., concepts, images, etc.).
(2) Elements can cohere or be incoherent.
(3) If two elements cohere there is a positive constraint between them. If they are incoherent there is a negative constraint.
(4) Elements are to be divided into ones that are accepted (cohere) and ones that are rejected (incoherent).
(5) A positive constraint between two elements can be satisfied either by accepting or rejecting both elements.
(6) A negative constraint between two elements can be satisfied by accepting one of the elements and rejecting another.
(7) The coherence problem consists of dividing a set of elements into accepted and rejected sets in a way that satisfies the most constraints.

That means for the clue task we start with the clues “Times” and “Inch.” Let us further assume that the concept “Times” activates among others a concept such as “Newspaper,” and “Inch” a concept such as “unit of measurements,” which results in a negative constraint between the activated concepts. A “feeling” of incoherence would occur. Providing additional information result in a coherent state until positive constraints between all the concepts are mutually activated.

Einstein struggled with incoherent pictures resulting from pieces of information which did not fit together. Re-connecting the given information with the new understanding of the importance of reference frame resolves the incoherence and consequently results in a coherent picture – positive and satisfied constraints.

However, Einstein’s thinking also shows the limitation of a pure constraint satisfaction account, because the solution is not always available in the initially activated search space. Sometimes it is necessary to overcome the given constraints to find novel coherent states (see stage 4 below).

Another question related to constraint satisfaction is how coherence could be implemented at a neuronal level. Lakoff and Johnson (1980) proposed a neural theory of metaphor (NTM) (Lakoff, 2009, 2014) which provides a detailed mechanism for coherence-building that has some relevance for our discussion. The following elements consolidate NTM (Lakoff, 2009):

• Neural groups. Small networks of neurons. Neurons can mutually be part of different groups.
• Spreading activation and Hebbian learning. Neural groups could inhibit or activate other groups. Important is the assumption that when two groups are simultaneously activated they become connected. This Hebbian learning principle (Hebb, 2005) might also be a key mechanism which is until now widely neglected in the domains of insight and intuition.
• Binding. There are three degrees of binding. Permanent (e.g., red ball – the red color is bound permanently to the round shape), conditional bindings – the binding is still permanent, but could have discrete forms (e.g., an object which is changing colors). Nonce – binding that happens on the fly.
• Fit. A node *A* fits better to a network *N* than to a network *N’* if *A* in *N* showed a higher overall number of neural binding than in *N’* (see p. 24).

**FIGURE 3:**
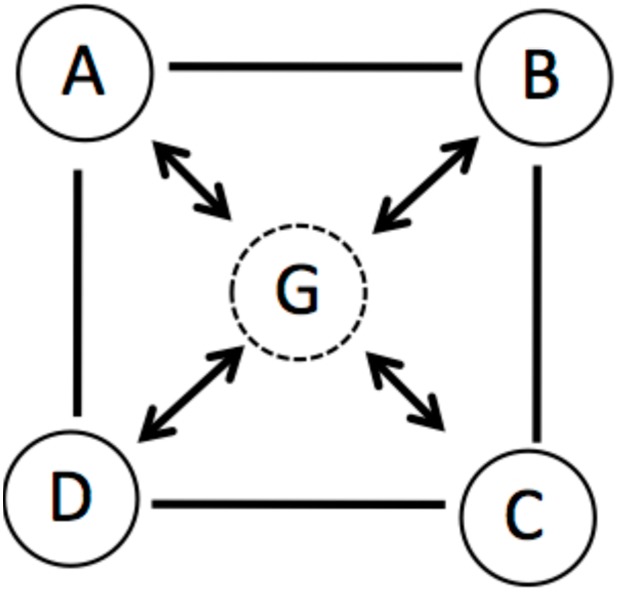
**Illustration of a Gestalt node which is tightly connected with the four other nodes**.

We postulate that a coherent state is closely related to Gestalt circuits. There are some nodes, e.g., *A*, *B*, *C*, *D*, and a Gestalt node *G* (**Figure 3**). If node *G* is firing, the nodes *A*, *B*, *C*, *D* are also firing. If a few nodes are activated and a threshold is surpassed, *G* is elicited. When *G* is inhibited, at least one of the other nodes is also inhibited. We propose that a Gestalt node could serve as coordinating hub which binds together information. The node *G* constrains the search space exciting A, B, C, D and inhibiting other nodes like E, F. NTM provides several mechanisms how distant concepts are linked together and how inferences could be drawn. Most important is the assumption that co-activation of remote concepts link those concepts and result in a coherent state.

We assume that for the clue example each single word could be seen as Gestalt node. The word co-activates several other words or meanings that are linked to this word. At the beginning (i.e., providing the first few clues) the clues excite remote and only partial and weak overlapping nodes. The more clues that are presented, the more likely it becomes that a particular node will be co-activated increasingly until it reaches the threshold to consciousness. The target word could be viewed as a new Gestalt node which binds distinct features of all the other clue words together.

The tight link between Gestalt perception, binding, and consciousness was shown by the detection of synchronized EEG signals (Singer, 1999; Engel and Singer, 2001; Jung-Beeman et al., 2004; Uhlhaas et al., 2006; Öllinger, 2009). An instructive example is the sudden recognition of an ambiguous figure, showing a Dalmatian dog sniffing at the ground (Tallon-Baudry and Bertrand, 1999). At the first glance the image seems a scrambled pattern of black and white colored patches. After a while the patches re-organize apparently out of nothing to an arrangement of meaningful objects. One explanation for this phenomenon emphasizes the importance of gamma-oscillations when viewers consciously recognize the Dalmatian dog. Tallon-Baudry and Bertrand (1999) proposed that this pattern stands for a binding process building a coherent picture from scrambled information.

## Stage 2: Coherence Detection

How does a person realize that a coherent state is reached? There are two intimately related concepts which might address this question. First, Topolinski and Strack (2009a,b,c), Topolinski et al. (2009), Topolinski and Reber (2010) showed that process fluency is closely related to a coherence state. As mentioned above coherent triads are processed more fluently than incoherent triads. Process fluency could be defined as the ease with which given information is processed by the cognitive system.

Process fluency could also be exploited as an indicator showing a transition in a person’s behavior while solving a series of problems. Haider and Frensch (1999), Wagner et al. (2004), Gaschler et al. (2013), Haider et al. (2013), Dietrich and Haider (2015) have been pursuing the idea that during learning of skills there are such transitions. They used for example the number reduction task (Wagner et al., 2004). In this task, participants were confronted by strings composed of three different digits. E.g., the string 1 1 4 4 9 4 9 4. There are two rules that have to be obeyed:

(1) *Same rule*: two identical digits reduce to the same digit. 1 1 → 1
(2) *Different rule*: two different digits reduce to the third digit 1 4 → 9

The task is to process the string stepwise from left to right. For the example given above 1 1 → 1. Then the task requires problem solvers to use the result from the first reduction and to take the next number from the string: 1 4 → 9; etc. The result of number reduction will be 9 for the string above. The strings were composed in a way that they either could be solved by this step-wise or sequential method, or much faster by realizing that there is a hidden rule, where the solution to the problem is already determined after the second attempt, since the sequence of the reduced digits is symmetrical [see Wagner et al. (2004) for the details of the task].

The number reduction task allows the moment of time to be determine when participants utilize the hidden rule. A sudden drop in the solution time is detectable, which could not be explained by step-wise learning process. Haider et al. (2013) postulated that after a large number of attempts implicit processes extract and detect the underlying regularity of the given sequences. This enters a processing shortcut resulting in a much higher process fluency. Such distinct behavioral changes could be realized consciously by the participants. The realization allows insight to be gained consciously into the symmetric nature of the response strings.

Another indicator that helps to realize a coherent state is the change of the affective state. This addresses the famous Aha! experience. The Aha! is described by a few dimensions, such as suddenness, positive affect, or the feeling of being right (Topolinski and Reber, 2010; Danek et al., 2013; Danek and Wiley, 2016). It seems conceivable that such changes could easily be detected by the problem solver and could lead to the re-evaluation of the problem-solving process.

It is important to note that an Aha! experience is not a proper predictor for the correctness of the solution (Koffka, 1935; Danek and Wiley, 2016; Salvi et al., 2016).

## Stage 3: Evaluation

At this stage the result of the coherence-building process is evaluated. The problem solver validates whether the solution fits the given requirements and meets the desired goal. The solution is either found and coherent or the result is incorrect, which necessitates a restart of the search.

Heider (1946) called a coherent state a *state of balance*. The given elements (information) fit together and there are no contradicting relations between the given elements. Following Heider’s account explains the need for coherence. Incoherence leads to tension within the system and there is a tendency toward a balance state. This might explain, why at the first place the cognitive system has a drive toward coherence. Heider’s field theoretical approach addressed the relations between persons and objects. Heider aimed at providing the determinants of social behavior and social perception. Beyond that, we propose that Heider’s account is generally applicable to situations where mutual relations of interdependent information are given. It provides a rule-based framework explaining the dynamics of coherence-building. Cartwright and Harary (1956, p. 266) summarized Heider’s account as follows.

Given a P-O-X unit consisting of a person P, another person O, and an impersonal unit X. The relations of each part of the unit are interdependent with each other. If P likes O and O is seen as responsible for X then there would be a tendency that P also would like X. This would be a balance state. If X has a negative relation with P then an imbalanced state results. In the person O the need arises to change the situation toward a balance state, e.g., by changing the relation between P and O from “like” to “not like.” A state of balance results Cartwright and Harary (1956) showed by a general graph theoretical account that Heider’s three elements approach can be extended to more complicated situations.

Following this account incoherence lead to the drive to search for a state of balance, and there is a schema that justifies that the deductions within the given information are mutually consistent. This implies the search for new relationships between the existing information driven by logical consistency with the existing information. This search process might to a great extent be unconscious, but will be shaped by the person’s attention, deliberations, prior knowledge, attitudes, and motivations.

The theory of balance has some similarities with Thagard and colleagues’ idea of constraint satisfaction (see above). An important question is how the cognitive system resolves existing conflicts.

Hélie and Sun (2010) proposed an elegant framework that provides a conflict resolution mechanism. Their explicit-implicit interaction theory (EII theory) assumes the parallel activation of *implicit processes* which are mainly associative. In contrast, *explicit processes* are driven by attention and characterized by more precise and distinct information processing. The explicit processes are predetermined by hard constraints. Processing of a new problem activates simultaneously the two systems. Conflict resolution is necessary, when no satisfying result is found. As a consequence, the results from both systems (implicit–explicit) will be integrated into one representation. This result is fed in as new input. The program cycles to the conflict resolution and integration cycle until the goal state is found.

The authors tested their model by a famous study on insight problem solving (Durso et al., 1994). Originally, Durso et al. (1994) introduced a graph theoretical approach. The approach combined the idea of semantic network analysis and the concept of restructuring (Ohlsson, 1984a,b). The goal of the study was to uncover participants’ underlying knowledge structures when solving an insight problem. Durso et al. (1994) asked participants to solve the following puzzle: “A man walks into a bar and asks for a glass of water. The bartender points a shotgun at the man. The man says, ‘Thank you,’ and walks out.” (Durso et al., 1994, p. 95). While solving the problem participants answered ‘yes’ and ‘no’ questions. The questions were intended to reveal the individual problem representation, e.g., question: “Was the man thirsty?” – answer: “No”. Afterward participants were asked to judge the relatedness of concepts of pairs (e.g., bartender, surprise). From this data semantic graphs were construed. In the next step, the authors compared the semantic graphs of solvers and non-solvers. It was found that solvers represented more likely direct connections between concepts which refer to the solution (e.g., surprise and remedy). Non-solvers focused more strongly on facts which were explicitly given (e.g., bartender and man). Solvers represented important aspects of the problem very early. Durso et al. (1994) concluded that the relatedness between certain concepts determines the likelihood for restructuring (see below, stage 4).

Given this finding Hélie and Sun (2010) modeled the hiccups problem with the connectionistic network (CLARION). CLARION’s explicit knowledge system was fed with answers to the yes–no questions. Initially, it mainly represented the given task instruction. The associations between concepts were randomly determined and built the implicit system. The degree of randomness was varied between conditions. The authors found that the higher the randomness score, the more likely is a graph structure which resembles the solvers’ structure actually found by Durso et al. (1994). Higher variation rates allowed a better conflict resolution that result in the desired solution.

Importantly, the authors suggested that higher randomness leads to more frequent remote and distant concept associations. Those associations are often incoherent with the given explicit knowledge representation. The conflict between the implicit and explicit representations might result in the generation of new and insightful hypotheses which help to solve the problem.

Conflict detection plays also an important role in the field of intuition research. Kahneman (2012) showed how misleading first intuitions could be. E.g., in the famous Linda problem a number of statements about a fictive person were given. Linda is 31 years old, outspoken, bright, single. She majored in philosophy. As a student she was deeply concerned with issues of discrimination, social justice, and also participated in anti-nuclear demonstrations (Tversky and Kahneman, 1983).

After reading the description participants were asked to choose the statement which seems more probable. (a) Linda is a bank teller. (b) Linda is a bank teller and is active in the feminist movement. Almost all participants opt for statement (b). The answer is wrong. Assuming the probability that Linda is a bank teller is 60% and the probability that she is active in the feminist movement is 70%. The product (conjunction) of both is 42% (0.6 × 0.7 = 0.42). The product is always smaller than each multiplier. Consequently, option (a) is the only correct answer. Tversky and Kahneman (1983) proposed that participants used an implicit (intuitive – system 1) heuristic which is biased toward option (b), because (b) seems more representative than (a). After a deliberate evaluation (system 2) it should become clear that (a) is the correct answer. In our discussion that means that a first coherent representation of the problem results from prior knowledge constraints or heuristics which restrict the evaluation process. Consequently, a conflict is detected between the apparent solution and the actual (logical) solution. In our model the participant would also commit an error, since an external feedback – whether the solution is correct – would be necessary at the evaluation stage to restart the process. Then the coherence building process could be restructured. We do not agree with Kahneman’s conclusion that intuitive processes are *per se* problematic. Moreover, there are alternative accounts which demonstrate how the conjunction fallacy could be explained (e.g., Tentori et al., 2013).

In contrast to Kahneman, Gigerenzer and Todd (2001a,b), Kruglanski and Gigerenzer (2011), Mega et al. (2015) assumed that intuitions help solving problems fast and frugal, e.g., facing the following question: “Which city has the better football team – Karlsruhe or Munich?” You do not know Karlsruhe, so you opt for Munich. The recognition heuristic (Gigerenzer and Todd, 2001a) helps to solve the problem. The idea is that uncertainty is reduced by relying on the ease of recognition. That means that a processing advantage indicates a potential solution to the problem. In our example, larger cities are more familiar. This corresponds to a higher likelihood of having a successful football team. However, Gigerenzer’s approach has also its limitations. Changing the cities in the above example and using the cities Nuremberg and Hoffenheim would result in a wrong solution by the recognition heuristic. Hoffenheim is fairly unknown but has the better football team than Nuernberg.

In sum, both the deliberate and the intuitive account need an evaluation process which justifies that the found solution is plausible and reliable. Both systems can provide erroneous results.

Generally, Kruglanski and Gigerenzer (2011) criticized (sensu Keren and Schul, 2009; Keren, 2013) that the dichotomy between an intuitive and deliberate system might be arbitrary. They proposed a rule-based account which relies in principle on if-then rules, as does our approach. The authors elaborated on this assumption and demonstrated that deliberate and intuitive judgments could be based on the same rules, as they demonstrated for the recognition heuristic (p. 100). They further assume that rules could be hardwired and explicit rules become implicit after training and expertise. The rule selection process is constrained by the task type. Certain heuristics do fit the task requirements others do not. Again, expertise and prior knowledge play an important role.

Thomson et al. (2015) provided a cognitive model that is based on the ACT-R framework to model intuitive-decision-making. ACT-R is a sophisticated production system. Productions consist of an IF statements (conditions) and a THEN part which represents an action. If a condition is matched the production system will execute an action. Productions could be newly learned, modified, or compiled. They are mostly explicit at the beginning of learning, and become implicit after repeated training. The ACT-R system is divided into an implicit and a declarative memory system (explicit), and has a goal stack which controls the flow of operations like a working memory. Information is stored in chunks. The strength of a chunk is determined by its recency and its frequency of retrieval (ease of recall). The authors assume that implicit memory content is activated by matching the given information. Spreading activation is pre-supposed and implemented by allowing associations between existing chunks. Attentional processes guide activation. The strengths of associations are determined by their co-occurrence in the past. The authors emphasized that intuition is a blend of consciously accessible and consciously inaccessible information. They suggest that retrieval processes are mainly unconscious, whereas declarative knowledge elements and the selection of heuristics and strategies are more deliberate and conscious.

Taken together the results of different fields show that intuitive and insightful problem solving could be modeled by rule-based accounts that entail similar properties (like implicit and explicit systems). Problem solving needs both systems to detect conflicts which drive the search for new associations. Eventually, the search results in new coherent state. However, there are situations where the building of new associations or the combination of implicit and explicit information is not enough. These situations require a deeper structural change, namely the restructuring of the search space.

## Stage 4: Restructuring as Coherence Building Process

The Gestalt psychologists (Wertheimer, 1923, 1959; Duncker, 1935; Koffka, 1935; Katona, 1940; Köhler, 1947) showed a major interest in answering the question under which conditions perceptual information is grouped to meaningful units. They identified that similarity, symmetry, and the proximity of perceptual elements affect the grouping process. For Köhler (1947), Wertheimer (1959) re-grouping (restructuring) of the given information was the major factor for productive or insightful thinking.

**FIGURE 4:**
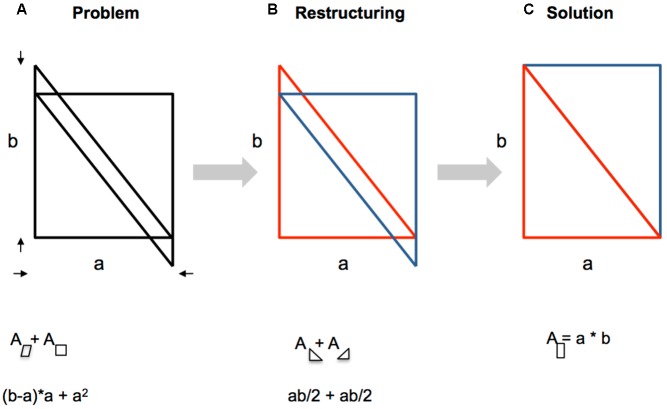
**Wertheimer’s Parallelogram-Square problem. (A)** Initial state. **(B)** Restructuring of lines. **(C)** Solution.

**Figure 4** illustrates the grouping dynamics by the Parallelogram-Square problem (Wertheimer, 1925). The task requires determining the total sum of the area of the parallelogram plus the area of the square, given “a” and “b” (**Figure 4A**). A beautiful solution entails that the given lines are restructured so that two rectangular triangles result (**Figure 4B**). Eventually, the triangles form a rectangle (new grouping, **Figure 4C**). Now, it is simple to determine the area “a” × “b”.

Within the field of insight problem solving constraints play a significant role (Isaak and Just, 1995; Knoblich et al., 1999). Ohlsson (1992, 2011) argues that a problem activates prior knowledge from long-term memory. The activated knowledge imposes constraints on the representation. It was demonstrated in several studies (Knoblich et al., 1999, 2001; Kershaw and Ohlsson, 2004; Öllinger et al., 2006, 2008, 2013, 2014; Danek et al., 2013, 2014; Kershaw et al., 2013) that self-imposed constraints caused the main source of problem difficulty. The relaxation of constraints leads to a new problem representation which allows for novel insights. There is a major transition from a state of “not knowing a solution” to a state of “knowing a solution” (Ohlsson, 2011; Danek et al., 2014).

**FIGURE 5:**
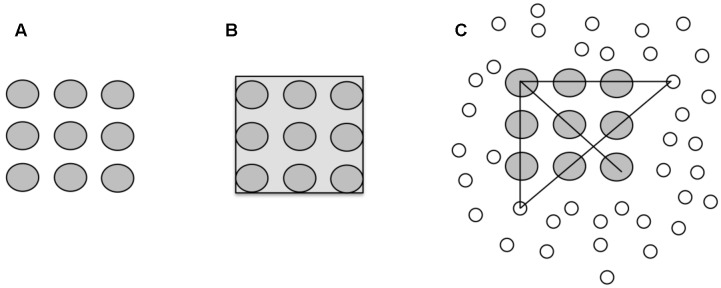
**(A)** The initial problem representation of the Nine-dot problem. **(B)** Perceptual coherence constrains the search space. **(C)** Enhanced search space after the perceptual constraint is relaxed.

It is important to note, that constraint satisfaction does not need to provide a solution. **Figure 5** shows the famous Nine-dot problem. The task is to connect the given nine dots by four connected straight lines, without lifting the pen, or retracing a line.

The Nine-dot problem proves to be extremely difficult. The common explanation claimed that a Gestalt-like perception of the given nine dots prevents drawing lines beyond the perceptual boundaries (Maier, 1930; Kershaw and Ohlsson, 2004; Öllinger et al., 2014). Importantly, and hardly recognized was the fact (Öllinger et al., 2014) that after problem solvers had relaxed the perceptual constraint an even larger search space resulted – adumbrated in **Figure 5C**. The scattered dots emphasize that after constraint relaxation (restructuring) lines could be drawn to arbitrary positions outside the boundaries of the nine dots. Consequently, it is not trivial to find the correct sequence of lines connecting all dots (Weisberg and Alba, 1981). Öllinger et al. (2014) showed that the concerted interplay between heuristics – restricting the search space – and constraint relaxation – expanding the search space – is sufficient to solve the problem.

In sum, restructuring allows problem solvers to search for the solution within a new search space. The larger search space enables the activation of new concepts. The new concepts could be integrated or build interrelationships with already existing concepts of the problem representation. It is necessary that the larger search space is restricted by constraints that guide the coherence-building process.

## Examples and Generalization

**FIGURE 6:**
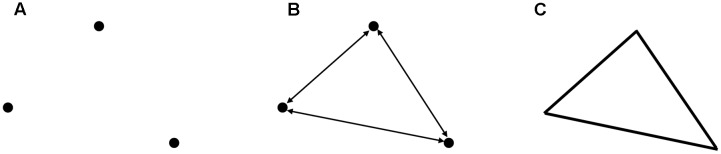
**Process of perceptual organization. (A)** Three arbitrary dots are given. **(B)** Interactions between dots unfold interrelationships and meaning. **(C)** Higher order meaning of a triangle could emerge.

In this section, we elaborate on the stage model. **Figure 6** shows an introductory example which illustrates the basic principles of coherence-building. First, three arbitrary dots were presented. According to our model, in stage 1 implicit processes spread activation and constrain the search space by prior knowledge. The dots “start” to build interrelationships with each other. At a neural level the dot pattern results in a synchronized spatial activation pattern which organizes the three dots into a unified representation (Koffka, 1935; Singer, 1999; Engel and Singer, 2001; Tallon-Baudry, 2003; Hebb, 2005).

Following Lakoff’s approach (**Figure 3**) the three dots will be connected via a Gestalt node which concerted the interplay and co-activation (Hebbs rule: “fire together wire together”) of the three dots. The Gestalt node coordinates the coherent state. The three dots build a triangle. The concept of a triangle (another Gestalt node) is associated with knowledge about triangles (form, rules, and theorems). This would be the result of stage 2. At a conscious level the recognition of a triangle occurs. At stage 3 the evaluation could focus on the question, whether this finding is significant, reliable, or interesting. However, it is not necessary and pre-determined that a triangle is recognized. Other coherent representations are conceivable and are mostly driven by the given task set, context, prior knowledge, and/or instructions, e.g., the three dots could also activate the concepts of a number (three) or trinity. Others will recognize the dots as representing individual subjects who have certain relationships – two of the dots seem to be linked closer. One seems to be more distant. In principle, a rather large number of coherent states are possible, all of them could be evaluated or further developed. Maybe the last example led the reader into a phase of restructuring which changes the coherence-building process (stage 4) from triangle to social domain.

Japanese haikus (von Müller, 2015) illustrate the dynamics of coherence-building in a more sophisticated field. Haikus are poems that have a well-defined phrase structure like in the famous haiku:

the stillness

penetrating the rock

a cicada’s cry

Basho (1644–1694)

Initially, reading Basho’s beautiful haiku word by word might seem confusing. It did not immediately become clear what is meant by the given words and how they are interrelated – a state of imbalance and conflicts might occur. After a few iterations through the phrases it is possible that new interrelationships between the concepts were elicited. First there is the image of a state of silence that is turned into a state of noise by a cicada’s cry. The contrast increases and is emphasized. It is alternating between stillness and noise, where both are so intense that even a rock is penetrated. This draws the picture of strong forces which almost hurt. Lastly, it is imaginable to assign different directions to the forces caused by noise and stillness. It seems that noise drills into the rock, whereas stillness corrodes the rock. The whole meaning unfolds from the presence of all three parts of the haiku and the constant re-interpretation (restructuring) of the different parts might result in a vivid image of the scene until a beautiful coherent representation (image) results.

Haikus might provide a rich source for new empirical research, e.g., to investigate in more detail how coherence-building is influenced when the order of the phrases is shuffled or words are replaced or substituted? Would it result in the same coherent image at the end or would it result in a distorted image which becomes meaningless?

**FIGURE 7:**
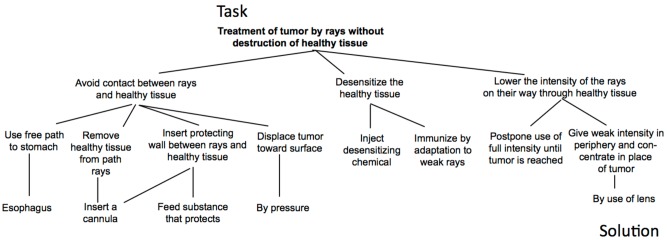
**Duncker’s thinking aloud graph.** The top node represents the task. Branching from the task three general approaches are illustrated which were further detailed.

Our final example is taken from the domain of insight problem solving. It is used to demonstrate how our model promotes a more detailed and elaborated view on problem representations of already well-known standard insight problems. We chose Duncker’s (1945) tumor problem: “Given a human being with an inoperable stomach tumor, and lasers which destroy organic tissue at sufficient intensity, how can one cure the person with these lasers and, at the same time, avoid harming the healthy tissue that surrounds the tumor?” Duncker used thinking aloud protocols as one of the first to uncover participants thought processes (Ericsson and Simon, 1993). **Figure 7** showed Duncker’s thinking aloud analysis of various solution attempts.

The most right-hand path in **Figure 7** shows an elegant solution to the problem. The solution requires superimposing rays of weak intensity at the tumor, so that the tumor is destroyed and the surrounding skin is not affected.

The tumor problem proved as reluctant to hints and analogical transfer (Gick and Holyoak, 1980, 1983), and was difficult to solve. For quite a long time it was unclear, what caused the difficulty of the problem.

**FIGURE 8:**
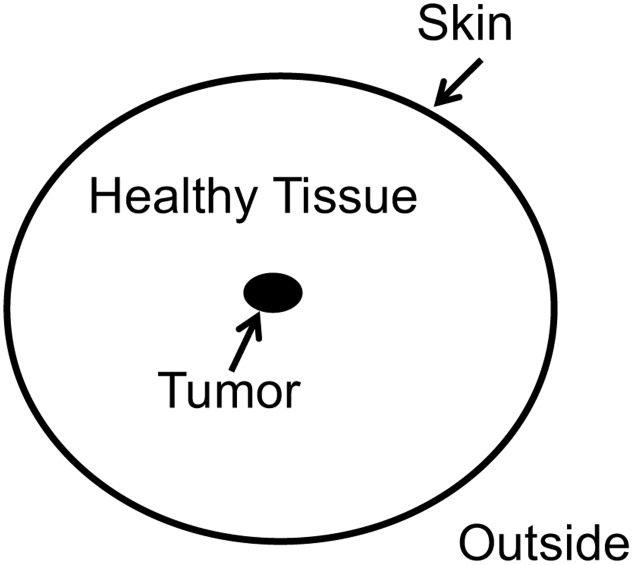
**Grant and Spivey’s tumor problem**.

Grant and Spivey (2003) provided participants with a sketch of the problem, such as **Figure 8**. In a first experiment, they recorded the eye-movement patterns. They analyzed the patterns of successful and un-successful problem solvers. They found that successful solvers more likely attended to the surrounding skin, whereas unsuccessful participants fixated on the tumor. Ingeniously, the authors run a second experiment with three conditions. In the animated skin condition, the skin was flickering. In the animated tumor condition, the tumor was flickering. In the third condition a static picture was presented (control condition). As expected the animated skin condition outperformed the two other groups (solvers: 67% animated skin; 33% animated tumor condition, 37% static control condition).

**FIGURE 9:**
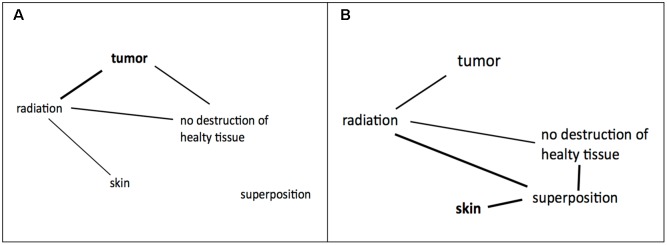
**(A)** Hypothetical initial representation of the tumor problem. **(B)** New coherent representation of the problem.

Duncker’s and Grant and Spivey’s findings suggest an initial representation of the tumor problem as depicted in **Figure 9A**. Initially, the given concepts were constrained by the importance of the tumor and did not integrate the remote concept “superposition” which is the key concept of the solution. After evaluation (stage 3) it becomes clear that a solution within this representation is impossible and a state of imbalance is achieved which increases the need to drive toward a state of balance. Restructuring (stage 4) is necessary which expands the search space. For the tumor problem restructuring requires a broad associative search with a high variation rate (Hélie and Sun, 2010). Importantly, the search process is not blind, but guided by constraints which are stated by the instruction and the goal representation which is strongly tied to the concept of “skin” (Dietrich and Haider, 2015). New associations are possible and a state of balance between the given concepts could be attained. Consequently, a coherent representation results which entails the solution to the problem.

According to Grant and Spivey’s (2003) finding skin becomes the driving concept which integrates superposition and leads to new interrelated concepts. Hebbian learning is elicited and leads to a new coherent representation which links the concepts tumor, destruction, laser, and superposition.

Currently, we realize a big gap between the empirical data which demonstrates effects according to varying experimental conditions and the underlying knowledge structures. We propose that our four-stage model allows for the pinpointing of knowledge structures. To do so, it is inevitable to validate hypothetical assumptions on potential problem representations by using quantitative measures which reveal the actual knowledge structure. We assume that the four-stage model might help to choose the appropriate means. In the next section we will summarize a few potential measures at the behavioral level.

## Measuring Coherence Building

Since the early work of cognitive psychologist (Newell and Simon, 1972) it has been a main goal to discover significant individual representations during the stream of problem solving. This also holds true for measuring coherence. How could the experimenter realize that a coherent state is achieved? Answering this question is important for the empirical test of our model. We assume that it is helpful to have different measures which could be assigned to the different stages of our model. We enlisted a few potential measures:

(1) Measuring processing speed. Measuring either the detection of coherence (stage 2) (Topolinski and Reber, 2010), or significant changes of the problem representation (Wagner et al., 2004) by faster response times or sudden drops of the processing speed.
(2) Implicit measures like lexical decision tasks or implicit association tasks to reveal which key concepts (Gestalt nodes) are activated during spreading activation by an individual at the beginning of the problem solving process or later on (stage 1–stage 4). This is crucial for learning more about the actual representations of the problem and potential changes during the time course.
(3) Semantic differentials or yes–no questions to assess whether a concept is part of the problem representation or not (Dayton et al., 1990; Durso et al., 1994) (stage 1, 2). This allows to generate knowledge graphs.
(4) A new approach to measure coherence would be to ask participants to draw explicit problem representations like in **Figure 9** by themselves after certain time intervals. The graph consists of the explicable basic concepts which are supposed to be important for the problem. The links between the lines reflect the association, and the thickness of the lines reflects the alleged strength of the concepts. The time series of individual representations reveals changes or mental impasses. Limitation of this approach would be that only explicable concepts will be represented, and the problem-solving process might be changed by this second task. This also holds true for (2) and (3).
(5) Eye-movement data could help to evaluate the importance of the given information for the problem-solving process (Knoblich et al., 2005).
(6) In the future, it is conceivable that new brain-imaging techniques will help to monitor coherent states in the brain. Recently Huth et al. (2016) were able to map natural speech to certain tiles of the cerebral cortex given fMRI data and sophisticated statistical methods.

We assume that a detailed understanding of coherence-building is the key to answer the questions when and why a biased or inappropriate representation leads to false intuitions or why problem solvers get stuck in an impasse. We also assume that to pinpoint the processes and knowledge structure it is crucial to decide whether a problem is solved with or without insight. Nowadays, we either rely on the weak and tautological assumptions that insight problems require insight, or we rely on subjective experience like indicating an Aha! (Öllinger and Knoblich, 2009).

## Discussion

We demonstrated that intuition and insight share some significant features and could be explained within a four-stage model. In both domains constraints play an important role. Constraints drive coherent states (Thagard, 2002), but also restrict the search space. Prior knowledge imposes rules and activates heuristics and problem solving strategies (Ohlsson, 2011). Intuition is in our understanding a result of a mainly automatic and implicit process which results from constraining processes and simple heuristics and rules which could lead to the solution or could be misleading.

A simple pattern matching mechanism guides the selection of competing rules or heuristics (Kruglanski and Gigerenzer, 2011). The selected rule determines the processing of the given information and determines the frame for the coherence building process. E.g., in our three dot example we showed that according to the selected rules, the three dots could cohere in a triangle, number representation, or social interactions. Spreading activation (Collins and Loftus, 1975) and the variation of combinatorial links between remote concepts are key features which help to come up with new coherent states of difficult problems (Hélie and Sun, 2010).

Coherence in this framework could be understood as state of balance (Heider, 1946), in which the concepts within the constraint representation have a consistent interrelatedness without conflicts. Such a state leads to a higher process fluency which causes detectable behavioral changes (Wagner et al., 2004; Topolinski and Strack, 2009c). Gestalt nodes (Lakoff, 2009) stand for the condensed meaning of the linked concepts and bind the given pieces of information together. Additionally, new meanings (links) could emerge by the binding processes. At the neural level, it seems plausible that Hebbian learning plays an important role and strengthens the connection between simultaneously activated concepts.

Our model extends Bowers et al. (1990) model in a few aspects. In contrast to Bowers’ model we assume a constraining process at the guiding stage in addition to spreading activation. Importantly, the accumulation of information is not a necessary criterion for a coherent state in our model. The coherence-building in our model is implemented by constraint satisfaction. The result is a balanced state. If such a state is reached, then the process fluency will increase, and cause behavioral changes (Wagner et al., 2004). Those changes foster that the coherent state surpasses the threshold to consciousness. Coherence-building is recursive, widely implicit and consists of conflict resolution and the integration of information. The process is affected and guided by attentional processes and deliberate thinking. We also emphasized the existence of a restructuring stage which overcomes already elicited coherent representations by changing the search space. This indicates a qualitative change in the problem-solving process. Still the constraint satisfaction process is active, but now more remote concepts could be integrated in a new representation. As we showed variation plays an important role to build those new associations (Fedor et al., 2017). In our understanding restructuring demarcates intuition from insight. Intuition could result from the realization of a coherent representation resulting in a hunch how a problem could be solved and accompanied by affective and cognitive processes. Whereas insight results from a restructured problem representation which allows a new and unusual solution to a problem which suddenly leads to a deep understanding of the given problem. That means intuition evaluates the coherence of the given information, whereas insight evaluates the result of restructuring.

## Open Questions and Limitations

An important premise that the four-stage model made is that constraint satisfaction and binding are the basic processes, one at a cognitive, the other a neural level. There are alternative accounts that question the idea of binding by synchrony (Hayworth, 2012) or provide alternative accounts for the combination of information, such as the latching mechanism provided by Amati and Shallice (2007), Song et al. (2014) or binding by convolution introduced by Thagard and Stewart (2011). We leave this question open to be answered by future work. We are positive about the fact that our model would also work with an alternative binding process.

Another open point is why the system tends to search for a coherent or state of balance? Related to this point is the question, is it possible that there are problems where imbalance is necessary to solve the problem? Furthermore, it would be helpful to determine at an individual level, which traits of characteristics of personality increase the probability of finding coherence.

The notion of a rule-based account is also questionable. This refers to the notion of dual systems. Dual system accounts in general differentiate between a fast, unconscious, unlimited, holistic (system 1) and a slow, deliberate, logical, restricted system (system 2) (Evans, 2008; Kahneman, 2012). Insight and intuition are often assigned to system 1 processes. For many years, there have been discussions, whether such two separate systems, modes or processes are necessary, plausible, well-defined and complete (Evans, 2008; Kruglanski and Gigerenzer, 2011; Kahneman, 2012; Evans and Stanovich, 2013; Mega et al., 2015).

In our line of argumentation, we followed Kruglanski and Gigerenzer (2011) who proposed a rule-based account in which the rules range between an explicit and implicit level. We think this account is also supported by the modeling accounts we reviewed above (Hélie and Sun, 2010; Thomson et al., 2015).

In contrast, Evans and Stanovich (2013) disagree with this proposal and argue that there are clear indicators for two systems. Most important would be the fact that only system 2 supports hypothetical thinking and showed heavy working memory load. Again, we are not in a position to resolve this discussion right now, but we think that our model might help to search for unified processes which vary in the processing stage.

In sum, we hope to demonstrate a more general model on insight and intuition which shows that insight and intuition are the two different sides of the same coin.

## Author Contributions

MÖ provided the review on grouping phenomena in psychology. AvM developed the idea on coherence building and provided the philosophical foundations.

## Conflict of Interest Statement

The authors declare that the research was conducted in the absence of any commercial or financial relationships that could be construed as a potential conflict of interest.
